# Knowledge distillation for efficient standard scanplane detection of fetal ultrasound

**DOI:** 10.1007/s11517-023-02881-4

**Published:** 2023-09-01

**Authors:** Jacopo Dapueto, Luca Zini, Francesca Odone

**Affiliations:** 1 MaLGa-DIBRIS, Università degli Studi di Genova, Genova, Italy; 2 Esaote S.p.A, Genova, Italy

**Keywords:** Standard scanplane detection, Fetal ultrasound, Knowledge distillation, Machine Learning

## Abstract

**Abstract:**

In clinical practice, ultrasound standard planes (SPs) selection is experience-dependent and it suffers from inter-observer and intra-observer variability. Automatic recognition of SPs can help improve the quality of examinations and make the evaluations more objective. In this paper, we propose a method for the automatic identification of SPs, to be installed onboard a portable ultrasound system with limited computational power. The deep Learning methodology we design is based on the concept of Knowledge Distillation, transferring knowledge from a large and well-performing *teacher* to a smaller *student* architecture. To this purpose, we evaluate a set of different potential teachers and students, as well as alternative knowledge distillation techniques, to balance a trade-off between performances and architectural complexity. We report a thorough analysis of fetal ultrasound data, focusing on a benchmark dataset, to the best of our knowledge the only one available to date.

**Graphical abstract:**

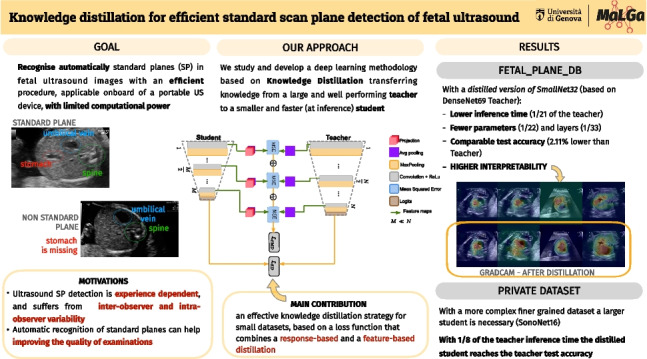

## Introduction

Abnormalities are one of the leading reasons for perinatal mortality in both industrialized and developing countries [1], thus mid-trimester fetal ultrasound (US) scans are carried out to provide accurate diagnostic information for the delivery of optimized prenatal care with the best possible outcomes for mother and fetus. During the obstetric US examinations, standard planes (SPs, sectional images containing key anatomical structures) are selected with care to compute biometric measurements to evaluate fetal growth and congenital malformations. They are also used to estimate the pre-birth weight and the gestational age of a fetus [1]. SPs are identified following international guidelines, promoted by scientific committees, so that images are obtained following the same protocols, in theory with the purpose of guaranteeing repeatability and reliability.

In clinical practice, the SPs selection based on the above-mentioned protocols is experience-dependent, cumbersome, and suffers from inter-observer and intra-observer variability. The situation is even more critical in some countries, especially in the developing world, where according to the World Health Organization (WHO) individuals with no formal training carry out ultrasound scans [1]. Hence, the *automatic identification of SPs* will facilitate more objective evaluations and overall workflow improvement.

**Fig. 1:**
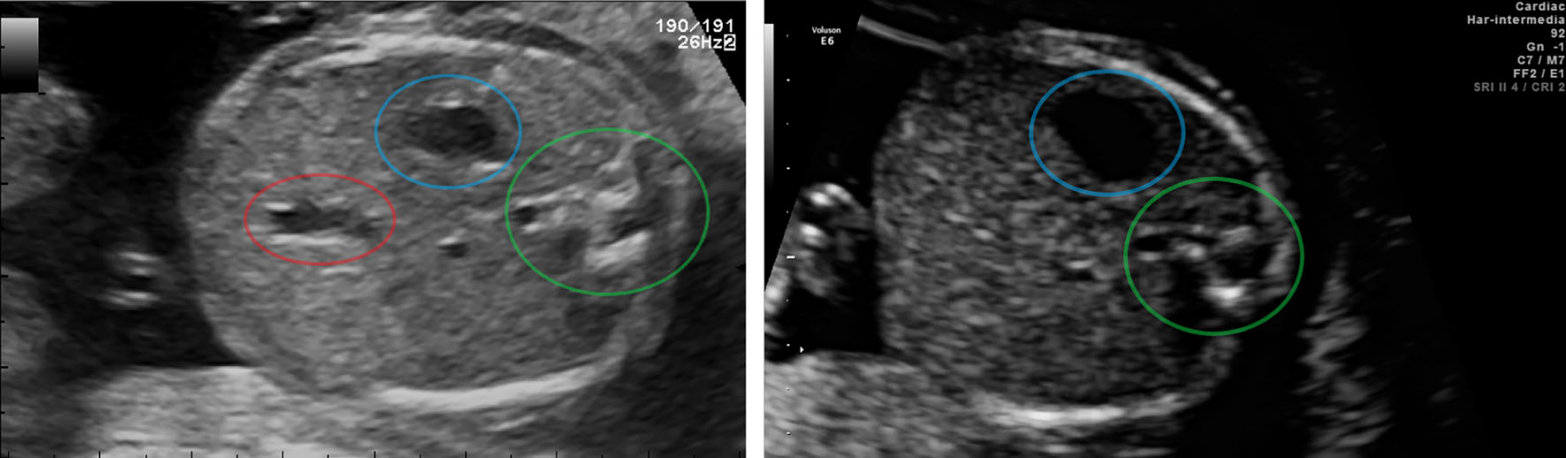
Examples of fetal *abdomens*, from [2]. Left: a standard plane compliant to fetal anomaly screening programme [1]: it includes the spine (*green*), the umbilical vein (*red*), and the stomach (*blue*). Right: a non-standard one, where the umbilical vein is missing

The task is challenging for several reasons, we highlight two important ones: *first*, the properties of US images [3]: low contrast, low signal-to-noise ratio, non-uniform acoustic densities and the presence of scattered noise. In addition, due to the high intra-class and low inter-class variations of US images, often non-SPs images are very similar to SPs [4, 5], so they are difficult to distinguish.

*Second*, in the identification of SPs of a given anatomical district, one should consider their difference with non-standard planes (non-SPs) of the same district may be minute — see Fig. 1. Indeed, SPs detection is different from the related task of US classification, since different images of the same anatomical district may be classified differently (standard or non-standard), while the goal of US classification is to associate an anatomical label to each example.

In this paper, we address the above-mentioned challenges by adopting a data-driven approach. Since the field is also suffering from a lack of data, data-driven solutions reduce the challenges related to data quality and variability, but raise a *third* issue: because of the legal rights to protect the privacy of patients, the example collection and annotation workload make data collection hard or sometimes even impossible. For this reason, medical images are valuable and often kept private: the literature is often reporting results based on private datasets [5–9], with a negative impact on methods benchmarking and reproducibility. In our work, we rely on the FETAL_PLANE_DB^1^, to the best of our knowledge, the first publicly available dataset of fetal US scanplanes [2].

**Fig. 2:**
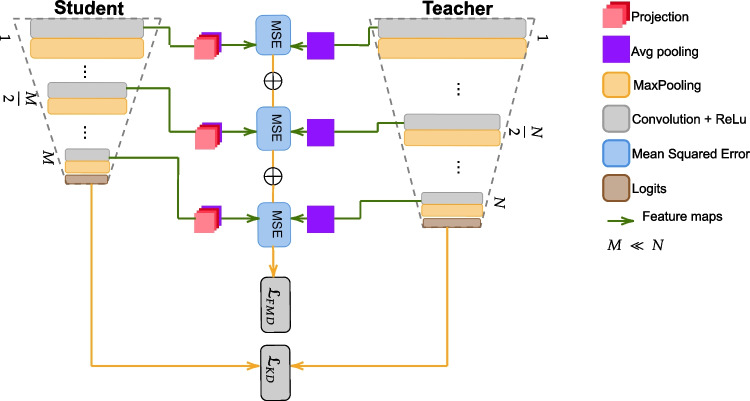
A sketch of the proposed knowledge distillation scheme, involving three convolutional layers of the student and teacher networks, and the final logits. Average pooling is employed to align the shapes of teacher and student feature maps, and a projection of student feature maps is learnt to reduce the gap between the capability of the two networks

Specifically, we focus on the problem of designing a computationally efficient network for SPs detection. Indeed, as a main functional requirement of our research, we require the network to be able to run onboard portable ultra-sound scanners, whose computational power is limited and fully dedicated to signal acquisition and processing. Portable units are cheaper and more flexible than traditional ultrasound machines and more appropriate for the market of developing countries, where automatic analysis functionality is particularly needed.

Following previous works in the literature, we formulate SPs detection as a multi-class classification task and investigate the usage of Knowledge Distillation (KD) [10] to keep the computational cost under control, transferring the generalization ability of a high-performing network (called *teacher*) into a second one (called *student*), with a two-fold benefit (1) reducing the dimension and the complexity of the model in terms of the number of layers and parameters; (2) mitigating the over-fitting effect due to the limited amount of training data. We propose a simple strategy distilling knowledge from Logits and from three hidden layers (see Fig. 2), resorting to a hand-crafted association.

To summarize, the *paper contributions* are as follows:

- A simple yet effective knowledge distillation strategy, based on a loss that combines a response-based and a feature-based distillation.
- A thorough evaluation of candidate choices for teacher and student models, taking into account a budget on data availability due to the specific application and inference time performance limits guided by the target portable US system.
- An efficient pipeline for standard SPs detection we assess on a fetal image classification benchmark to provide measurable evidence of the potential of the approach in the clinical domain.

## Methods

### Related works

**SPs detection.** With the development of deep learning, general-purpose CNNs have been applied in many medical applications, including US classification [2, 11, 12]. In the case of US classification, a recent study assesses the performances of several architectures [2], showing the potential of large CNNs in this domain. It does not discuss CNNs limitations due to the stringent computational requirements of the device, and the limited availability of data, and this lack of information motivates our analysis, while the architectures considered by the authors are the candidates for our teacher models.

Other works learn the relationship between 2D plane images and 3D volumes [13], exploit the temporal relation between the frames in video clips [11, 14], other completely based their approach on the identification of the ASs [7]. The limited availability of data in the field, pushed the researchers to improve the generalization ability of the models with different strategies, for instance designing ad hoc features[15], adopting multi-task learning classifying and localizing ASs [8, 16], and including clinical prior knowledge [8, 16] increasing the burden of data acquisition and annotation, or integrating an *attention mechanism* so that the predictions are performed thanks to local features at different scales [9].

Fewer, *ad hoc* architectures are also available in the literature: SonoNet is a family of CNNs specifically designed to solve the detection of SPs in real-time during the US scanning [5].

**Knowledge distillation.** KD is a class of methods from the deep learning literature, whose goal is to control the complexity of a network by distilling knowledge from a large teacher model to a smaller student model mainly comparing either the logits [17] or the outputs of the hidden layers of both models [18–21], after the distillation only the student will be used for inference. In the literature, hidden layer associations are either done by hand before training [18–20] or learnt during the optimization of the student [21]. As it will be clarified in the following, we adopt the former approach.

Knowledge distillation has been previously applied to SPs detection in ultrasound imaging to classify echo cine series into 12 standard views [22], and to detect the abdomen, femur and head from fetal images [6]; both methods proposed memory-efficient student models, with no specific focus on the inferring time. Both approaches take into account a richer input, with respect to our work: [22] analyze US clips, while [6] complement US images with relevant information acquired by an eye tracker.

### Proposed methodology

We formalize SPs detection as a multi-class classification task, where each image is associated with one of *n* predefined classes and the set of classes may include SPs and non-SPs of the same anatomical district, and for this reason, some classes may be very similar to one another.

The task is complex and fine-grained, for this reason, small and specific architectures may not be sufficient. At the same time, large architectures may not be applicable, to the stringent computational requirements. We adopt a KD strategy, where we distil the knowledge from a more complex general purpose *teacher model* to a simpler more specific *student model*.

In the method we propose, we combine two alternative KD approaches: a first approach, BasicKD, using the output of the last layer before the softmax, called *Logits*, so the student is trained to produce Logits equal to those generated by the teacher [17].

A second intermediate KD considering the outputs of the hidden layers of the CNN, comparing the feature maps generated by the student with those of the teacher, involving compatible layers, otherwise the optimization will lead to suboptimal solutions. Here, we follow a hand-crafted association, motivated by the fact, we are interested in distilling knowledge between architectures with the same hierarchical structure, including layers with similar semantic meanings.

Figure 2 provides a sketch of the methodology we propose.

The distillation loss we propose combines a response-based and a feature-based part, the hyperparameter $\beta$ controls the importance of features:

1 $$\begin{aligned} \mathcal {L} = \mathcal {L}_{KD} + \beta \mathcal {L}_{FMD}. \end{aligned}$$

$\mathbf {\mathcal {L}_{KD}}$: it is a loss computed on the Logits, as the linear combination of two components, whose relative weight is controlled by a hyperparameter $\lambda \in [0,1]$.

2 $$\begin{aligned} \mathcal {L}_{KD}= \lambda \mathcal {L}_{SL} + (1 - \lambda )\mathcal {L}_{logits} \end{aligned}$$

The mismatch between the output of the student model and the ground-truth label is computed with the function *H* representing the cross-entropy loss:

3 $$\begin{aligned} \mathcal {L}_{SL}=H(softmax(a_s), y_r) \end{aligned}$$

$a_s$ is the student network logit and $y_r$ the ground-truth label.

The difference between the probability distributions generated by the *softmax* is computed with the *Kullback–Leibler* (*KL*) divergence loss.

4 $$\begin{aligned} \mathcal {L}_{logits}=\tau ^2 KL(softmax(\frac{a_t}{\tau }), softmax(\frac{a_s}{\tau })) \end{aligned}$$

A hyperparameter $\tau$, called *temperature*, controls the softening of the signal arising from the output of the teacher network; $a_t$ and $a_s$ are the logits of the teacher and student networks, respectively. More details on this loss can be found at [17] (Sec. Distillation) where the Cross-Entropy loss is adopted, but it has been proved it is equivalent to the KL divergence ([23] Sec. 2).

$\mathbf {\mathcal {L}_{FMD}}$: it is computed on the intermediate feature maps, as a *hand-crafted* version of the adaptive method proposed in [21]. As shown in Fig. 2 the knowledge transfer is performed taking into account only three convolutional layers with *RELU* activation function for the student and for the teacher: the *first*, the *intermediate*, and the *last* layers of the teacher as associated with the corresponding layers of the student, through an appropriate feature map loss, combining Mean Square Error (MSE) losses:

5 $$\begin{aligned} \mathcal {L}_{FMD} = MSE_1 + MSE_2 + MSE_3. \end{aligned}$$

Since the two networks should be of different size scales and based on different architectures, in order to align the shapes of the features between the teacher and the student, an average pooling [24] is performed to both student and teacher’s features map. A projection including a stack of convolutional layers of the size $1\times 1$, $3\times 3$, and finally $1\times 1$ is further applied to the student’s features to reduce the gap between capabilities of the networks.

### Implementation details

To estimate the above-mentioned hyperparameters, we perform *grid search* on the following ranges: $\lambda \in \{i(0.1) \mid i \in \mathbb {Z}, 1 \le i \le 9 \}$, $\tau \in \{i \mid i \in \mathbb {Z}, 1 \le i \le 10 \}$, $\beta \in \{ 1, 5, 10, 100, 200, 400, 800 \}$, weight decay regularization $wt\in \{5e^{-5}, 5e^{-6}, 1e^{-5}$, $1e^{-6}, 1e^{-7}, 1e^{-8}\}$, learning rate $lr\in \{0.01, 0.001, 0.0001, 0.00005\}$.

We train the models with 10% of the training set as a validation, and the models achieving maximum validation accuracy, in a given epoch, are selected.

Because of the limited size of the datasets available, we adopt the following data augmentation:

1. Randomly *flipped horizontally*, with a probability of 50%;
2. Randomly *flipped vertically*, with a probability of 50%;
3. *Gamma Correction* with randomly selected gamma between 0.3 and 1.7;
4. Normalized so that the values of the pixels range from 0 to 1;
5. *Zoom* with a random factor between 80% (zoom out) and 120 (zoom in);
6. Random rotation in a range between −10$^\circ$ and 10$^\circ$.

Gamma correction is performed to mimic different gain settings used during the examination to overcome US attenuation and different tissue echogenicity, while the other transformations encode the possible settings the sonographer could choose to visualize US images on the US scanner and the possible fetal position in the placenta.

The tables report the *validation accuracy* (VA) used for the model selection, and the *balanced* validation/test accuracy (VAB, TAB). The *balanced* accuracy is computed as the mean of the diagonal of the *confusion matrix* and it is not considered during the optimization phase.

As teachers, we considered state-of-the-art architectures of different kinds, like DenseNet and ResNet, characterized by the potential to reach high accuracy but a huge amount of parameters and layers. For the student, we choose the SonoNet family, which is characterized by the absence of fully-connected layers, replaced with convolutions also for the final prediction before the softmax.

## Results

In this section, we report and discuss the results we obtain on the FETAL_PLANE_DB, benchmark, available online [2].

The dataset is composed of over 12400 images from 1792 patients, acquired on 6 different machines by several different operators with similar experience. They are divided into 6 classes (Table 1): four of the most widely used fetal anatomical planes (**abdomen**, **brain**, **femur** and **thorax**), the mother’s **cervix** (used for prematurity screening) and a general category to include any **other** less common image planes (not necessarily fetal or maternal), further dataset statistics can be found in [2] (Table 1). The authors already divided into the *training* and *test* *subsets* to simplify the comparison with the state-of-the-art, containing 7129 and 5271 images, respectively. The *validation* is 10% of the training set, as in [2].

**Table 1:** FETAL_PLANE_DB: cardinality of the entire dataset

Anatomical plane	N. patients	N. images
Fetal abdomen	595	711
Fetal brain	1082	3092
Fetal femur	754	1040
Fetal thorax	755	1718
Maternal cervix	917	1626
Other	734	4213

**Table 2:** FETAL_PLANE_DB: state-of-the-art CNNs assessed as *teacher models* (number of Layers and Total parameters from [25])

Network	Layers	Total parameters	Sec/Frame (*ms*)	VA	VAB	TAB
VGG16 [26]	21	14,717,766	14.9	95.17%	93.74%	93.35%
MobileNet [27]	88	3,235,014	35.4	94.60%	94.88%	93.55%
0.75-MobileNet	88	1,837,590	36.0	94.31%	93.66%	93.59%
0.25-MobileNet	88	220,086	35.6	87.50%	81.77%	83.94%
Inception-v3 [28]	313	21,815,078	104.0	95.88%	95.75%	95.02%
ResNet50 [29]	177	23,600,006	71.9	95.17%	95.52%	94.75%
ResNet101 [29]	347	42,670,470	132.4	96.30%	95.68%	94.69%
ResNet152 [29]	517	58,383,238	196.5	95.31%	94.83%	94.45%
DenseNet121 [30]	429	7,043,654	145.4	96.02%	95.89%	94.66%
DenseNet169 [30]	597	12,652,870	204.7	96.44%	96.27%	95.13%
SonoNet64	53	14,864,350	25.8	96.59%	96.17%	95.10%

Seconds required for the forward stage through the network for 1000 samples and a batch size of 1 with Quadro RTX 5000

### Choice of a teacher model

We first aim to find the best performing existing model on the data in hand, to be the *teacher* model.

We start by reproducing the results presented in [2]: in Table 2, we report our performances, higher than the ones in [2] (average improvement of 2.13%) as we change *augmentation* and make the learning rate *lr* and the weight decay *wd* parameters *decay exponentially*, starting from an initial value $5e-5$ as weight decay and $1e-4$ as learning rate, updating every 100 steps and a decay rate of 0.9. The number of layers and parameters clearly state these architectures are not appropriate for the task at hand; 0.25-MobileNet is suitable in terms of the number of parameters but its performances are not sufficient for our purpose. DenseNet169 remains the one achieving the best performances, as reported in [2], and for this reason, we consider it a valid teacher model.

We include in our analysis an architecture from the SonoNet family, SonoNet64, which has a comparable size and performance to DenseNet169. It will also be considered in the following, as a teacher candidate.

**Table 3:** FETAL_PLANE_DB: candidate *student* models assessment

Network	Layers	Total parameters	Sec/Frame (*ms*)	VA	VAB	TAB
SmallNet16	18	71,334	9.4	95.88%	93.60%	90.11%
SmallNet32	18	282,950	9.4	95.59%	93.43%	91.29%
SonoNet8	53	234,966	25.3	96.16%	95.14%	93.48%
SonoNet16	53	933,646	25.4	96.44%	93.82%	93.23%
SonoNet32	53	3,722,238	25.1	95.59%	95.15%	91.77%

**Table 4:** FETAL_PLANE_DB, dataset: knowledge distillation. *Improvement*: difference of balanced test accuracy with respect to the corresponding baseline student model. The distillation may improve ($\uparrow$) or worsen ($\downarrow$) the performances

Teacher	Student	KD Method	VA	VAB	TAB	Improvement
DenseNet169	SonoNet32	BasicKD	97.30%	95.76%	93.89%	$\uparrow$(2.12%)
DenseNet169	SmallNet32	BasicKD	97.15%	94.13%	92.05%	$\uparrow$(0.76%)
SonoNet64	SmallNet32	BasicKD	97.15%	96.44%	92.77%	$\uparrow$(1.48%)
DenseNet169	SmallNet32	SemCKD	94.46%	93.01%	89.27%	$\downarrow$(2.02%)
SonoNet64	SmallNet32	SemCKD	96.02%	95.14%	91.58%	$\uparrow$(0.29%)
DenseNet169	SmallNet32	Ours	96.87%	96.39%	93.02%	$\uparrow$(1.73%)
SonoNet64	SmallNet32	Ours	96.73%	97.03%	92.80%	$\uparrow$(1.51%)

### Choice of a student model

The smaller architectures from Table 2 do not appear to be suitable student models because the inferring time is still quite high. We evaluate a selection from the family of SonoNet architectures instead, training them with a fixed learning rate of $1e-3$, a batch size of 16 and ADAM optimizer. The results are in Table 3. While the dataset is rich enough for bigger networks to get better performances, here, we are interested in smaller networks in terms of layers (to decrease the time of the prediction) and in terms of filters (i.e., number of parameters). We select SmallNet32 as our student model, and baseline for the forthcoming experiments because it represents a trade-off between size (w.r.t. parameters and layers), accuracy and computational performances.

### Knowledge distillation

Table 4 reports the results comparing our approach to KD with BasicKD [17] and SemCKD [21]. The best performances are achieved with DenseNet169 as teacher and our hand-crafted KD strategy. In the case of BasicKD the validation accuracy reaches 97.15%, also surpassing the performance of the teacher, but the same behavior is not appreciated in the balanced validation accuracy. SemCKD is instead ineffective, with a degradation of the results w.r.t. the baseline student model. It appears that the automatic association of the layers is possibly too challenging (the attention mechanism needs to learn additional parameters) for the limited amount of training data available. Moreover, the teacher and student architecture structures are very similar, therefore the data-driven association of SemCKD appears to be unnecessary. Instead, our hand-crafted association reports very promising performances, both in terms of validation and testing accuracy, with an increase of the balanced accuracy of SmallNet32 of about 1.73% with respect to the reference student model.

We also report the performance of SonoNet32 obtained with BasicKD, leading to higher test accuracy. However, this model cannot be a suitable candidate because its dimensionality is incompatible with our needs (in particular the number of parameters).

To support the choice of a three-tier association of our hand-crafted approach, we perform the grid-search with 2 and 4 correspondences, where the Teacher is DenseNet169 and while the Student is SmallNet32. In this case

- 2-layers associates the *first* and the *last* layers;
- 4-layers associates the first, the *median*, the last layer, plus a layer in the middle of the second half of the model.

We report the results of the selected models in Table 5 where we notice the three-tier association is the one achieving the best performances for all the metrics. Considering the size of the Student, it is not appropriate to associate a higher number of layers.

For an interpretation of the KD benefits, we compare the GradCam [31] heatmaps of the SmallNet32 model from Table 3 with the heatmaps of the best model in Table 4 (thus, we interpret the results before and after knowledge distillation). Notice that, even though SonoNet has been designed with a weakly supervised localisation embedded into the architecture, here we, use a generic GradCam for an effective comparison between the two outputs.

**Fig. 3:**
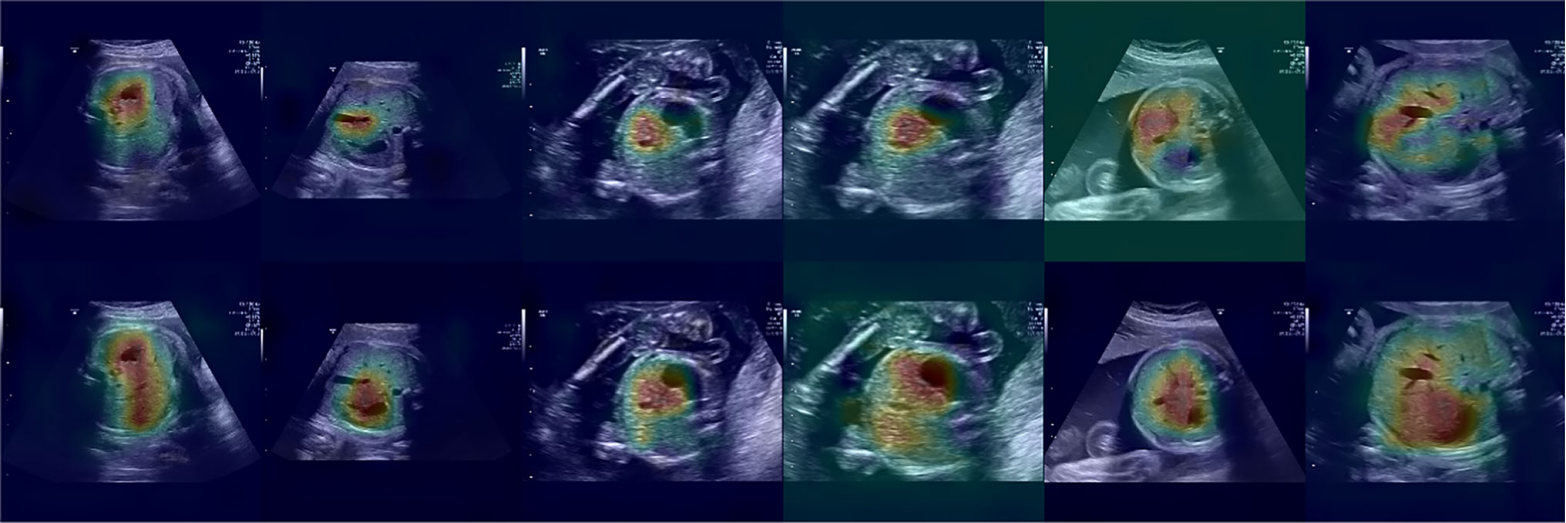
Heatmaps obtained via gradcam of 6 random abdomen samples from FETAL_PLANE_DB, test set [2], before (top) and after (bottom) KD (see text)

Figure 3 compares the same abdomens generated from the baseline model and the distilled one. The abdomen plane is denoted by the presence of the spine, the umbilical vein and the stomach (Fig. 1). On the first row (prior KD), the model focuses on one structure only (either the vein or the stomach). On the second row (after KD) the model captures both the umbilical vein and stomach bubble. Both models never take the spine, possibly because the spine is always present in abdominal images. The distilled model seems to rely on wider regions and it allows to generate features maps with a higher discrimination power.

**Fig. 4:**
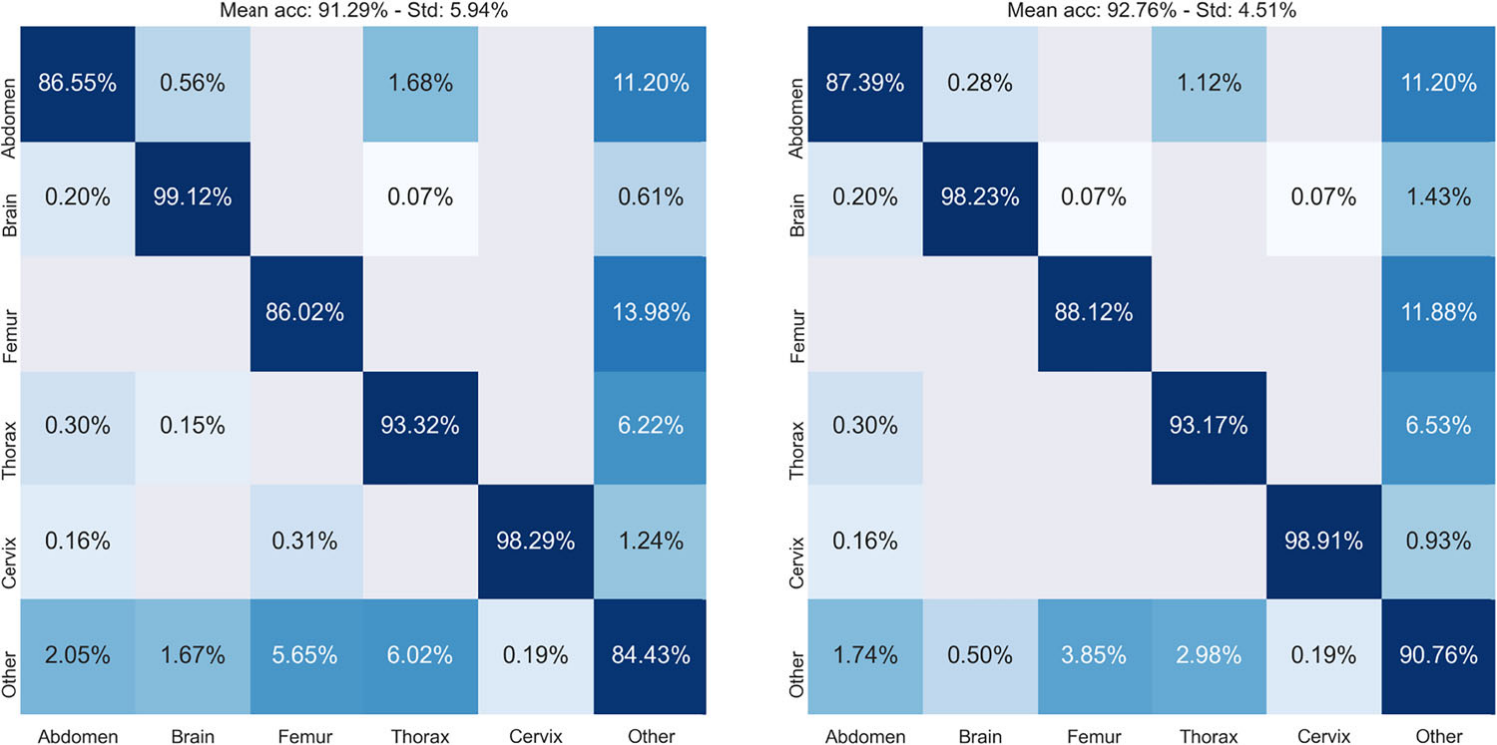
Confusion matrix of distilled SmallNet32 on the FETAL_PLANE_DB, test set [2]. Left: before the knowledge distillation. Right: after distillation. The colours scale is logarithmic to better highlight the misclassifications

Finally, Fig. 4 shows the confusion matrices obtained by SmallNet32, again before and after knowledge distillation. Each row represents the instances in a ground-truth class, while each column represents the instances in a predicted class. Most misclassifications involve the class *Other*, which includes non-SPs of several anatomical districts. The errors are mitigated by knowledge distillation.

## Discussion

The experimental analysis we carried out confirms that, with an appropriate choice of a distillation strategy, we may transfer knowledge from larger well-performing networks to smaller ones. The latter can be installed on a portable device, allowing us to achieve high efficiency (since they rely on few parameters and require a low inference time) while maintaining a good detection performance.

**Table 5:** FETAL_PLANE_DB, dataset: knowledge distillation with hand-crafted association. The column *Associations* reports the number of layers of the teacher and student used to perform the distillation

Teacher	Student	Associations	VA	VAB	TAB
DenseNet169	SmallNet32	2	95.59%	95.66%	92.88%
DenseNet169	SmallNet32	3	96.87%	96.39%	93.02%
DenseNet169	SmallNet32	4	94.74%	94.09%	92.13%

We achieve an *inference time improvement* with our best distilled student (SmallNet32) requiring $\frac{1}{21}$ of the time needed by the reference teacher network, DenseNet169 (while the model proposed in [22] is only $\frac{1}{6}$). As for the space occupancy of the student network, it contains about $\frac{1}{22}$ of the teacher’s parameters and $\frac{1}{33}$ layers.

Moreover, with our hand-crafted distillation strategy, we obtain a *test accuracy improvement* with respect to the student model prior to distillation The improvement mainly consists of a reduced misclassification on the class “Other”, the most challenging one because of its internal variability and its appearance overlap with several classes. This is due to the way the dataset was built [2], since only images complying with minimum quality requirements were selected by the clinicians, while low quality and inappropriate anatomical planes (cropped or badly taken) were labelled as *Other*, together with images containing calipers.

Apart from “Other”, the most challenging class that received a benefit from the distillation procedure is “Abdomen”, where most of the errors are mis-classifications with “Thorax” (see Fig. 4 ). Besides this, we could not appreciate any interpretable pattern in our errors.

It should be noted that SPs detection would be better addressed if we dispose of a dataset with several non-standard classes, one per anatomical district of interest. Unfortunately, to date, a dataset with these characteristics is not available.

If we compare the results of the student after distillation with the original teacher model, we notice a small performance degradation of $2.11\%$ with SmallNet32, while SonoNet32 (Table 4) architecture, whose inference time is $\frac{1}{8}$ of DenseNet169, leads to a higher test accuracy just with BasicKD, but a much higher dimensionality with respect to SmallNet32. Notice that classical CNNs like VGG16 have a similar inference time, a much larger spatial occupancy (they have a very high number of parameters, higher than DenseNet169), and lower performances.

## Conclusions

In this paper, we addressed the detection of SPs, with the objective of implementing the method onboard of a portable US scanner. First, we explored the performances of general state-of-the-art CNNs but the design of such networks is incompatible with the system’s computational requirements. The family SonoNet, explicitly designed for the detection of SPs, was tested looking for a small and fast but effective combination. The obtained results are encouraging, but the performances of the smallest models (e.g., SmallNets) are not competitive with the state of the art. To reduce this gap, we identified knowledge distillation strategies: KD allows to reduce the size of the network while maintaining satisfactory performances. We transferred the capabilities from a teacher to a student thanks to the logits and features maps of intermediate convolutional layers; we found out a manual association leads to better results, possibly because the available data are not enough to learn an automatic and optimal association. The best solution we obtained is a distilled SmallNet32, leading led to the best trade-off between speed and performance on the benchmark dataset.

An interesting direction for future works would be to further push the performances of the models with additional expert knowledge, similarly to [6]. The outcome of this research will be the development of software tools to help inexperienced operators with US acquisition tasks, highlighting SPs of interest as the examination takes place and recording them automatically during the exam. From a practical point of view, it would be highly beneficial to obtain this automatic classification on board the ultrasound device, as a guideline to the operator during scanning. The outputs would also be available for an offline analysis carried out by an expert ultrasonographer.

## Data Availability

FETAL PLANE DB is a publicly available dataset. For the ethics declaration, we refer to the original datasets publication.

## Footnotes

1 https://doi.org/10.5281/zenodo.3904280 Version: 1.0.
