# Correction to: Knowledge distillation for efficient standard scanplane detection of fetal ultrasound

**DOI:** 10.1007/s11517-023-02930-y

**Published:** 2023-09-13

**Authors:** Jacopo Dapueto, Luca Zini, Francesca Odone

**Affiliations:** 1 MaLGa-DIBRIS, Università degli Studi di Genova, Genova, Italy; 2 Esaote S.p.A, Genova, Italy

**Correction to: Medical & Biological Engineering & Computing**

**https://doi.org/10.1007/s11517-023-02881-4**

The original version of this article unfortunately contained a mistake.

The graphical abstract displayed in the published online version is incorrect. The correct image is shown below:
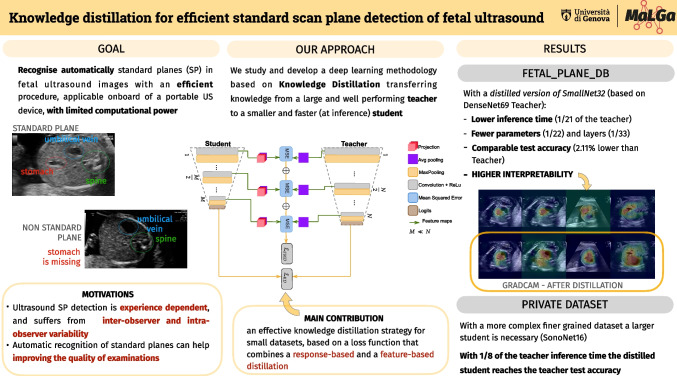


The original article has been corrected.

